# Adding a new piece to the puzzle of Cosmocercidae evolutionary relationships: genetic characterization of *Aplectana pella* parasitic in *Osteocephalus cabrerai* from Amazon Region

**DOI:** 10.1590/S1984-29612025007

**Published:** 2025-02-03

**Authors:** Jorge Kevin Silva Neves, Gabriel Lima Rebêlo, Adriano José Silva Félix, Scott Lyell Gardner, Arnaldo Maldonado, Carlos Eduardo Costa-Campos, Francisco Tiago Vasconcelos Melo

**Affiliations:** 1 Laboratório de Biologia Celular e Helmintologia “Profa. Dra. Reinalda Marisa Lanfredi”, Instituto de Ciências Biológicas, Universidade Federal do Pará – UFPA, Belém, PA, Brasil; 2 Harold W. Manter Laboratory of Parasitology, University of Nebraska-Lincoln – UNL, Lincoln, NE, United States; 3 Laboratório de Biologia e Parasitologia de Mamíferos Silvestres Reservatórios, Instituto Oswaldo Cruz – IOC, Fundação Oswaldo Cruz – FIOCRUZ, Rio de Janeiro, RJ, Brasil; 4 Laboratório de Herpetologia, Departamento de Ciências Biológicas e da Saúde, Universidade Federal do Amapá – UNIFAP, Macapá, AP, Brasil

**Keywords:** helminths, nematodes, molecular, phylogenetic, helmintos, nematódeos, molecular, filogenia

## Abstract

*Aplectana* comprises species of gastrointestinal helminths commonly found parasitizing amphibians and reptiles worldwide. However, most species of the genus are described based only on morphological traits. During helminthological surveys, we found nematodes identified as *Aplectana pella* parasitizing the hylid frog *Osteocephalus cabrerai* from the same locality as the original description. We provided the first nucleotide sequence of ribosomal gene 18S rDNA for *Aplectana pella* and established the species' phylogenetic position between representatives of Cosmocercidae. A pairwise genetic comparison between *A. pella* and its congeners revealed a low genetic divergence. We found that our sequences clustered with species of *Cosmocerca,* reinforcing the hypothesis that representatives of the genus *Aplectana* do not form a monophyletic group.

## Resumo

O gênero *Aplectana* inclui espécies de helmintos gastrointestinais comumente encontrados parasitando anfíbios e répteis, mundialmente. No entanto, a maioria das espécies do gênero foi descrita com base apenas em caracteres morfológicos. Durante expedições helmintológicas, foram encontrados nematódeos identificados como *A. pella* parasitando o anuro hilídeo *Osteocephalus cabrerai* na mesma localidade da descrição original. Foi fornecida a primeira sequência de nucleotídeos do gene ribossomal 18S rDNA de *Aplectana pella* e se estabeleceu a posição filogenética da espécie em relação aos representantes de Cosmocercidae. Comparações genéticas entre *A. pella* e seus congêneres revelaram uma baixa divergência genética. Foi encontrado que as sequências estudadas agrupam com espécies de *Cosmocerca*, reforçando a hipótese que representantes do gênero *Aplectana* não formam um grupo monofilético.

## Introduction

Nematodes of the genus *Aplectana* Railliet & Henry, 1916 (Nematoda: Cosmocercidae) are common parasites of the gastrointestinal tracts of amphibians and reptiles globally. To date, 58 species have been described, with most occurring in anurans from the families Bufonidae and Leptodactylidae (Campião et al., 2014; Vieira et al., 2020; Santos et al., 2023).

In recent years, molecular approaches, including DNA sequencing of specific genes, have been shown to be useful in estimating phylogenetic relationships among species in the family Cosmocercidae (Alcantara et al., 2022; Rebêlo et al., 2023). However, the identification of species of *Aplectana* from the Neotropical region continues to be based on morphological traits (Chen et al., 2021a). Currently, available genetic data of *Aplectana* spp. include only *Aplectana hylambatis* (Baylis, 1927) from South America, *Aplectana chamaeleonis* (Baylis, 1929) from Africa, *Aplectana dayaoshanensis* Chen, Ni, Gu, Sinsch & Li, 2021; *Aplectana macintoshii* (Stewart, 1914) and *Aplectana xishuangbannaensis* Chen, Gu, Ni & Li, 2021 from Asia (Chen et al., 2021a, b).

During a long-term study of the parasites of vertebrates of the Neotropics, we collected specimens of *Aplectana* from the large intestine of the frog *Osteocephalus cabrerai* (Cochran & Goin, 1970) from the Brazilian Amazon. An initial morphological analysis of the nematodes from these frogs suggested that it represented a new species, from which we successfully obtained molecular data. However, further morphological analysis using scanning electron microscopy allowed us to identify it as a previously described species *Aplectana pella* Santos, Borges & Melo, 2023.

*Aplectana pella* was described by Santos et al. (2023) from the intestines of the rusty tree frog, *Boana boans* (Linnaeus, 1758). However, at that time, this species was characterized using light and scanning electron microscopy. Thus, this study aims to provide a genetic characterization of *A. pella* and assess its phylogenetic relationship with other representatives of the familiy Cosmocercidae.

## Material and Methods

During a helminthological survey in the Amazon basin, 84 specimens of *O. cabrerai* were collected between May 2019 and March 2022 in the Beija-Flor Brilho de Fogo Extractive Reserve (0°47 '30.6' N; 51°58 '42.1' W), located in the municipality of Pedra Branca do Amapari, Amapá state, Brazil. The amphibian hosts were identified following the methodology of Pedroso-Santos et al. (2019).

After capture, frogs were euthanized and standard field morphological measurements were taken, and all specimens were necropsied for helminthological examination. All internal organs were removed and placed in Petri dishes with saline solution (NaCl 0.9%), dissected separately, and the contents of the organs and the organs themselves were examined under a stereomicroscope (LEICA EZ4). All helminths found were rinsed in saline solution, killed with heated 70% alcohol, and preserved in the same solution at room temperature; a collection tag with the host collecting number was placed in each vials. Methods generally followed Gardner et al. (2012).

The prevalence, mean intensity, and mean abundance of parasite infections are reported according to Bush et al. (1997). For morphological analysis, nematodes were hydrated in distilled water, cleared in Amann's lactophenol 20%, mounted on temporary slides, and examined with a microscope (Olympus BX41, Olympus Corp., Tokyo, Japan) coupled with a drawing tube (without zoom adjustment). Two male specimens were post-fixed in 1% Osmium tetroxide (OsO_4_), dehydrated in an increasing ethanol series and critical-point dried in Carbon dioxide (CO_2_). Specimens were mounted on metallic stubs, coated with gold-palladium and examined with a scanning electron microscope Vega3 (TESCAN, Brno, Czech Republic) in the Laboratory of Structural Biology, Biological Sciences Institute, Federal University of Pará (UFPA), state of Pará, Brazil.

For molecular analysis, a male was transferred to microtubes containing 100% ethanol and stored in a freezer at −20 °C. We extracted genomic DNA using NucleoSpin Tissue (Macherey-Nagel, Düren, Germany) according to the manufacturer’s instructions. The SSU rDNA gene (18S) was amplified using the protocol and primers described in Gomes et al. (2015).

The resulting amplicons were visualized on 1.5% agarose gel electrophoresis with GelRed Nucleic Acid Stain (Biotium, Hayward, California, USA) on an ultraviolet light transilluminator. PCR products were purified through Illustra GFX PCR DNA and Gel Band kit (GE Healthcare, Chicago, Illinois, USA) according to the manufacturer’s instructions and sequenced using the BigDye Terminator v3.1 Cycle Sequencing kit (Applied Biosystems, USA). Amplicons were sequenced on an Applied Biosystems™ 3730 DNA Analyzer at the DNA Sequencing Platform of the Oswaldo Cruz Foundation (RPT01A/PDTIS/FIOCRUZ).

Contiguous sequences were assembled in Geneious 7.1.3 software and deposited in Genbank (NCBI, 2024). We used the BLAST search to confirm the genetic proximity with other sequences of Cosmocercidae available in the Genbank database. The 18S rDNA datasets were aligned and trimmed using Muscle in Geneious 7.1.3 software. We obtained the saturation-substitutions index of each aligned matrix using the software DAMBE 5. Levels of genetic divergence were estimated using the MEGA 11.0 software package. The Akaike Information Criterion (AIC) via the jModelTest software determined the most appropriate evolutionary nucleotide substitution model. Sequence alignments were then subjected to Maximum Likelihood (ML) and Bayesian Inference (BI) analysis in RAxML 8.2.12 and MrBayes 3.2.7a softwares, respectively. Both analyses were carried out in CIPRES Science Gateway. Only nodes with posterior probabilities greater than 90% were considered well-supported. Maximum Likelihood inference (ML) was implemented, and estimates of the level of robustness of the tree estimations were done using bootstrap analysis through 1,000 repetitions, and only nodes with bootstrap values greater than 70% were considered well-supported.

The trees were visualized and edited in FigTree v1.4.4 software. We used *Ichtyobronema hamulatum* (Moulton, 1931) (access number: KY476351) and *Dichelyne grandistomis* (Ferraz & Thatcher, 1988) (access number: KX752094) as two separate outgroups. The sequences were selected based on Chen et al. (2021a, b), Svitin et al. (2023) and Santos et al. (2024), and poorly aligned sequences were excluded from the analysis. Detailed information on the nematode species included in the molecular analysis is provided in Supplementary Table S1.

## Results and Discussion

**Figure 1:**
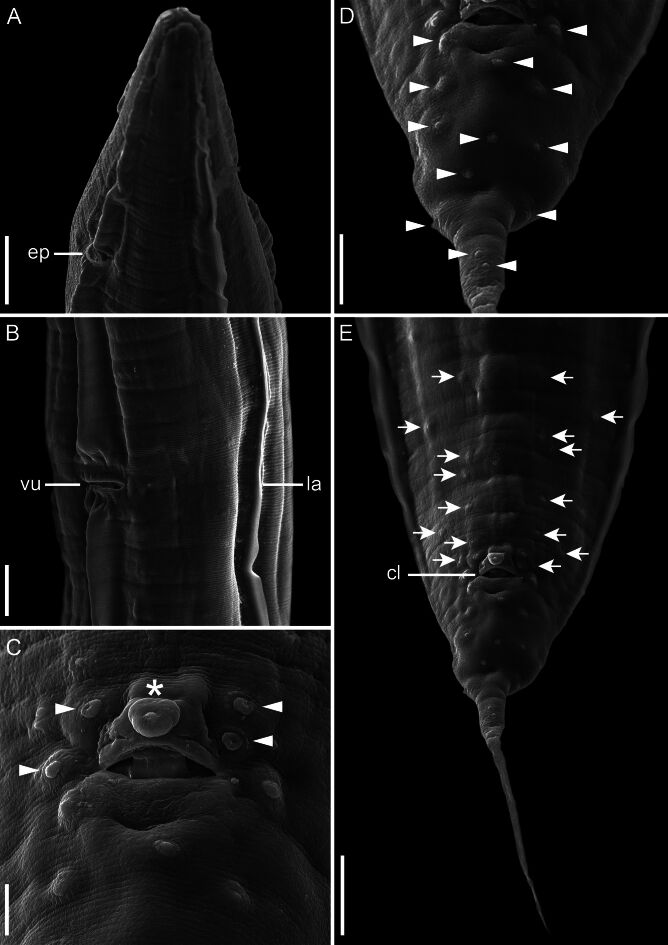
Scanning electron micrographs of *Aplectana pella* from *Osteocephalus cabrerai*. **(A)** Male, anterior end, lateral view; **(B)** Female vulva, ventral view; **(C)** Male, details of caudal papillae; **(D)** Male, distribution of postcloacal papillae; **(E)** Posterior end of male showing somatic papillae. Arrows: somatic papillae; arrowheads: cloacal papillae; asterisk: unpaired papillae. Abbreviations: cl, cloaca; ep, excretory pore; vu, vulva; la, lateral alae. *Scale bars*: A= 100 μm; B, E= 50 μm; C= 10 μm; D= 25 μm.

Prevalence, mean intensity, and mean abundance of parasite infections in the frogs studied here were 27.4%, 4.3 ± 3.9 (range 1–15) and 1.18 ± 2.8, respectively. All parasites were adults and were found in the large intestine. The morphology of the specimens analyzed here are identical to the original description and all measurements overlap the range of variation reported for *A. pella* (see Santos et al., 2023) (Table 1). We observed that the number and distribution of caudal papillae (two ventral precloacal papillae pairs near anterior cloacal lip; one adcloacal pair; five postcloacal pairs; one single unpaired papilla situated on anterior cloacal lip), gubernaculum absent, vulva equatorial and spicule lengths (see Figure 1C-D) are the same as indicated by Santos et al. (2023).

**Table 1:** Morphometric data from *A. pella* of *O. cabrerai* and *A. pella* from the original description.

Characters	*A. pella*		*A. pella*	
	Host: *Osteocephalus cabrerai*		Host: *Boana boans*	
	Present study		Santos et al. (2023)	
	Males	Females	Males	Females
	(n =10)	(n =10)	(n =7)	(n =10)
Total length (mm)	2.58 (2.10–3.13)	4.29 (2.86–5.52)	2.63 (2.3–2.9)	3.5 (2.3–4.3)
Maximum width	300 (253.3–386.6)	378.9 (263.1–512)	-	361.5 (289–436)
Body width at oesophago-intestinal junction	236 (208–280)	288.9 (192–350)	229.5 (205–251)	262.6 (178–306)
Body width at nerve ring	119.1 (96–138.6)	146.7 (120–180)	-	-
Body width at excretory pore	184.3 (160–216)	228.5 (162.6–269.3)	-	-
Lateral alae to anterior extremity	139.2 (93.3–186.6)	215.9 (173.3–280)	-	-
Lateral alae to posterior extremity	275.7 (226.6–317.3)	284.2 (250.6–325.3)	-	-
Oesophagus total length	468 (417–530.6)	593.7 (482.6–680)	464.3 (416–502)	565.5 (529–594)
Oesophagus in % of body length	18.2 (16.5–20.5)	14.3 (11.6–18)	17.6 (16.6-19.3)	16.5 (13.3–25.9)
Pharynx length	33.7 (26.6–40)	39.8 (32–50)	35 (27–42)	43.8 (32–50)
Pharynx width	27.7 (24–37.3)	34.4 (26.6–40)	28 (26–32)	32.3 (26–37)
Corpus length	320.5 (280–360)	414.4 (328–480)	302.7 (269–330)	377.1 (336–413)
Corpus width	44.2 (37.3–53.3)	50 (40–60)	-	-
Isthmus length	20.5 (10.6–29.3)	18.7 (13.3–25)	39 (32–45)	39.2 (32–50)
Isthmus width	29 (27–37)	37.3 (29.3–50)	-	-
Bulb length	93 (80–104)	120.7 (93.3–150)	87.5 (77–98)	105.5 (96–117)
Bulb width	113 (96–133)	145.9 (109.3–181.3)	109.2 (101–122)	129.5 (114–144)
Nerve ring from anterior end	184.2 (154.6–213.3)	218.7 (192–285)	194.5 (173–226.5)	213.9 (178–245)
Excretory pore	334.6 (266.6–392)	393.3 (277.3–490.6)	328.6 (312–344)	404.1 (349–453)
Tail length	327.6 (285.3–354.6)	373.2 (306.6–415)	307.7 (256–344)	358.1 (321–394)
Tail length in % of body length	12.8 (10.5–14.6)	9 (7–12)	11.7 (9.4–13.3)	10.3 (8.2–14.5)
Tail width	107.1 (74.6–149.3)	135.4 (90.6–213.3)	-	-
Spicules	110 (80.5–142.8)	-	106.5 (104–111)	-
Vulva to anterior end (mm)	-	2.29 (1.72–2.72)	-	1.63 (0.6–2)
Vulva in % of body length	-	54.5 (48.5–63.9)	-	45.3 (25.4–53.1)
Egg length	-	63.9 (45.7–78)	-	59.6 (54–67)
Egg width	-	40.9 (30.1–51.9)	-	36.5 (33–43)

*Aplectana pella* was originally described in the hylid frog *Boana boans* from Amapá state, Brazil (Santos et al., 2023). Our specimens were discovered as parasites of another arboreal hylid, *O. cabrerai* from the same locality, with similar parasitological descriptors (prevalence: 27.38% in *O. cabrerai* vs. 25% in *B. boans*; mean intensity: 4.3 in *O. cabrerai* vs. 6.5 in *B. boans*; mean abundance: 1.18 in *O. cabrerai* vs. 1.63 in *B. boans*), indicating that both hosts occupy similar ecological niches (Neves et al., 2024).

We obtained a fragment of 789pb long for the 18S rDNA gene from *A. pella*. The BLAST search revealed a sequence closely related to those of Cosmocercidae species available in the NCBI database. The alignment of the gene upon trimming to the shortest sequence length resulted in 747bp and included 16 species distributed across four genera: *Aplectana* (five sequences), *Cosmocerca* Diesing, 1861 (four sequences), *Cosmocercoides* Wilkie, 1930 (four sequences), *Nemhelix* Morand & Petter, 1986 (one sequence) and the outgroups. The best-fitting nucleotide substitution model identified was TIM3 + G (gamma shape parameter a = 0.0340; lnL = -1761.1172). Xia’s test provided no evidence for substitution saturation in the data matrix.

Pairwise genetic comparison between congeners of *A. pella* revealed the lowest genetic distance from *A. chamaeleonis* (1.09%), followed by *Aplectana hylambatis* (1.63%), *A. xishuangbannaensis* (3.59%), and *A. dayaoshanensis* (3.74%) (see Supplementary Table S2). This molecular marker is a well-conserved gene that evolves slowly (Koubková et al., 2008). Thus, our study reinforces the idea that the 18S rDNA region is a good marker for discriminating among genera and a good candidate for phylogenetic studies.

**Figure 2:**
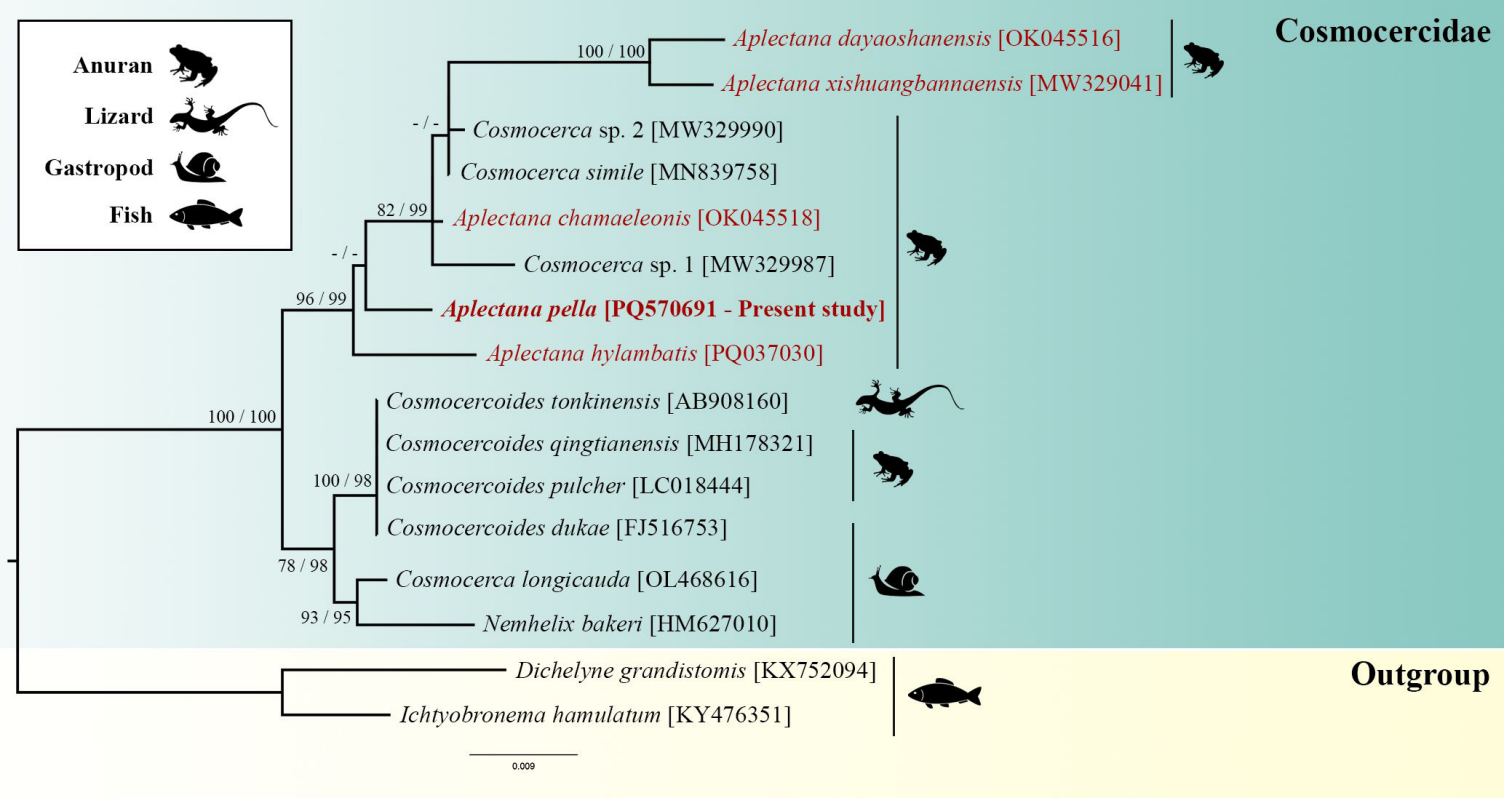
Maximum likelihood topology based on 18S rDNA using *Ichtyobronema hamulatum* and *Dichelyne grandistomis* as outgroup. GenBank accession numbers are indicated next to species names. Numbers beside the nodes represent support value by bootstrap for maximum likelihood analysis and posterior probabilities for Bayesian analysis, respectively (bootstrap scores >70 and posterior probabilities >90). Branch-length scale bar indicates number of substitutions per site.

Our phylogenetic trees obtained using Maximum Likelihood (ML) and Bayesian Inference (BI) revealed similar topologies. The sequences of Cosmocercidae (100 bootstrap and 100 posterior probability) formed two large groups (Figure 2). The first was composed of *Cosmocercoides* spp., *Cosmocerca longicauda* (Linstow, 1885) and *Nemhelix bakeri* Morand & Petter, 1986 (78 bootstrap and 99 posterior probability).

The second group included separate branches of *A. hylambatis* and *A. pella* (96 bootstrap and 99 posterior probability). Also, *A. pella* represents a sister group to a paraphyletic group that includes species of *Aplectana* and *Cosmocerca* (35 bootstrap and 64 posterior probability). Our results reinforce that *Aplectana* is a non-monophyletic genus, consistent with findings from previous studies (Chen et al., 2021a, b; Svitin et al., 2023; Santos et al., 2024). In a recent study, Santos et al. (2024) suggested that the geographic distribution of the species influenced the evolution of *Aplectana*. However, in the present work, the two sequences from Brazilian specimens did not cluster together, rejecting this hypothesis.

Traditional systematic studies have historically supported the evolutionary hypothesis that *Cosmocerca* is closely related to *Cosmocercoides*, mainly because those two genera share the presence of ornamented papillae in male caudal region (Wilkie, 1930; Chabaud, 1978). However, our results support recent phylogenetic studies that found *Cosmocerca* to be a non-monophyletic genus, closely related to *Aplectana* (Figure 2) (Chen et al., 2021a, b; Harnoster et al., 2022; Ni et al., 2022; Svitin et al., 2023; Tsuchida et al., 2023; Santos et al., 2024).

*Cosmocercoides* spp. did appear in our tree as monophyletic and clustered as a sister group to *N. bakeri* + *Cosmocerca longicauda* (Figure 2). This result has also been observed in previous studies (Saito et al., 2021; Harnoster et al., 2022; Ni et al., 2022; Svitin et al., 2023; Tsuchida et al., 2023; Santos et al., 2024). However, some studies suggest that the authors who deposited the sequence of *C. longicauda* misidentified the species (Svitin et al., 2023; Félix et al., 2024). Thus, until now, only *Cosmocercoides* have been found parasitizing snails, and due to the high genetic divergence observed among *C. longicauda* and other *Cosmocerca* spp. we also reinforce that this sequence should be considered a representative of the genus *Cosmocercoides.*

This study represents the first phylogenetic analysis including *A. pella*, that showed this genus as non-monophyletic. Therefore, further molecular-phylogenetic studies are necessary to understand better the evolutionary relationships of *Aplectana* species, particularly in the Neotropical region where significant gaps in the genetic database and taxonomic status exist among the species. We also emphasize the importance of combining detailed morphological and molecular studies with more representatives of the genus to improve our knowledge about the diversity and phylogenetic relationships of Cosmocercidae.

## Supplementary Material

Supplementary material accompanies this paper.

Table S1. Nematode species, hosts, localities, GenBank accession numbers, and references used in phylogenetic analyses.

Table S2. Pairwise genetic divergence levels (%) of the 18S gene among sequences of nematodes of the family Cosmocercidae. Names, GenBank accession, and values numbers are provided in the table.

This material is available as part of the online article from https://doi.org/10.1590/S1984-29612025007
