# Supplementary material for: Adding a new piece to the puzzle of Cosmocercidae evolutionary relationships: genetic characterization of Aplectana pella parasitic in Osteocephalus cabrerai from Amazon Region
Source: Rev Bras Parasitol Vet. 2025 Feb 3;34(1):e018124. doi: 10.1590/S1984-29612025007 (PMC11801249; doi:10.1590/S1984-29612025007)
Supplement: Table S1. [file rbpv-34-1-e018124-suppl01.pdf]

Table S1. Nematode species, hosts, localities, GenBank accession numbers, and references used in phylogenetic analyses.

Families	Species	Host	Locality	Accession numbers	Reference
**Cosmocercidae**	*Aplectana pella* Santos, Borges and Melo, 2023	*Osteocephalus cabrerai* (Cochran and Goin, 1970)	Brazil	XXXXX	**Present study**
	Aplectana hylambatis (Baylis, 1927)	*Pithecopus azureus* (Cope, 1862)	Brazil	PQ037030	Santos et al. (2024)
	Aplectana dayaoshanensis Chen, Ni, Gu, Sinsch and Li, 2021	*Hylarana spinulosa* (Smith, 1923)	China	OK045516	Chen et al. (2021a)
	Aplectana chamaeleonis (Baylis, 1929)	*Hyperolius kivuensis* Ahl, 1931	Rwanda	OK045518	Chen et al. (2021a)
	Aplectana xishuangbannaensis Chen, Ni, Gu and Li, 2021	*Polypedates megacephalus* Hallowell, 1861	China	MW329041	Chen et al. (2021b)
	Cosmocerca longicauda (Linstow, 1885)	Snail	-	OL468616	Unpublished
	Cosmocerca simile Chen, Zhang, Feng and Li, 2020	*Bufo gargarizans* Cantor, 1842	China	MN839758	Chen et al. (2020)
	Cosmocerca sp. 1	*Hoplobatrachus chinensis* (Osbeck, 1765)	China	MW329987	Chen et al. (2021b)
	Cosmocerca sp. 2	*Bufo melanostictus* (Schneider, 1799)	China	MW329990	Chen et al. (2021b)
	Cosmocercoides dukae (Holl, 1928)	*Deroceras panormitanum* Lessona, and Pollonera, 1882	USA	FJ516753	Ross et al. (2010)
	Cosmocercoides pulcher Wilkie, 1930	*Bufo formosus* Boulenger, 1883	Japan	LC018444	Tran et al. (2015)
	Cosmocercoides qingtianensis Chen, Zhang, Nakao, and Li, 2018	*B. gargarizans*	China	MH178321	Chen et al. (2018)
	Cosmocercoides tonkinensis Tran, Sato, and Luc, 2015	*Acanthosaura lepidogaster* Cuvier, 1829	Vietnam	AB908160	Tran et al. (2015)
	Nemhelix bakeri Morand and Petter, 1986	Snail	-	HM627010	Saito et al. (2021)
**Quimperiidae (Outgroup)**	*Ichtyobronema hamulatum* (Moulton, 1931)	*Lota lota* (Linnaeus, 1758)	Russia	KY476351	Sokolov & Malysheva (2017)

**Cucullanidae (Outgroup)**	*Dichelyne grandistomis* (Ferraz & Thatcher, 1988)	*Oxydoras niger* (Valenciennes, 1821)	Brazil	KX752094	Pereira & Luque (2017)

References cited in Table S1

Chen H-X, Ni X-F, Gu X-H, Sinsch U, Li L. Morphology, genetic characterization and phylogeny of *Aplectana dayaoshanensis* n. sp. (Nematoda: Ascaridida) from frogs. *Infect Genet Evol* 2021a; 96: 105123. https://doi.org/10.1016/j.meegid.2021.105123.

Chen H-X, Gu X-H, Ni X-F, Li L. Description of a new species of *Aplectana* (Nematoda: Ascaridomorpha: Cosmocercidae) using an integrative approach and preliminary phylogenetic study of Cosmocercidae and related taxa. *Parasit Vectors* 2021b; 14(1): 165. https://doi.org/10.1186/s13071-021-04667-9.

Chen H-X, Zhang L-P, Feng Y-Y, Li L. Integrated evidence reveals a new species of *Cosmocerca* (Ascaridomorpha: Cosmocercoidea) from the Asiatic toad *Bufo gargarizans* Cantor (Amphibia: Anura). *Parasitol Res* 2020; 119: 1795–1802. https://doi.org/10.1007/s00436-020-06687-3.

Chen H-X, Zhang L-P, Nakao M, Li L. Morphological and molecular evidence for a new species of the genus *Cosmocercoides* Wilkie, 1930 (Ascaridida: Cosmocercidae) from the Asiatic toad *Bufo gargarizans* Cantor (Amphibia: Anura). *Parasitol Res* 2018; 117: 1857-1864. https://doi.org/10.1007/s00436-018-5877-8.

Pereira FB, Luque JL. An integrated phylogenetic analysis on ascaridoid nematodes (Anisakidae, Raphidascarididae), including further description and intraspecific variations of *Raphidascaris* (*Sprentascaris*) *lanfrediae* in freshwater fishes from Brazil. *Parasitol Int* 2017; 66(1): 898–904. https://doi.org/10.1016/j.parint.2016.10.012.

Ross JL, Ivanova ES, Spiridonov SE, Waeyenberge L, Moens M, Nicol GW, et al. Molecular phylogeny of slug-parasitic nematodes inferred from 18S rRNA gene sequences. *Mol Phylogenet Evol* 2010; 55(2): 738–743. https://doi.org/10.1016/j.ympev.2010.01.026.

Saito T, Hayashi K, Hayashi K, Akita Y, Une Y, Kuroki T, Shibahara T, et al. Morphological observation and first molecular characterization of Grassenema procaviae Petter, 1959 (Cosmocercoidea: Atractidae) in the stomach of Cape hyrax (Procavia capensis) raised in a zoo in Japan. Parasitol Int 2021; 84: 102385. https://doi.org/10.1016/j.parint.2021.102385.

Santos PS, Silva ICO, Ferreira VL, Tavares LER, Paiva F, Pereira FB. First genetic characterisation and phylogenetic position of Aplectana hylambatis (Nematoda: Cosmocercidae), infecting Pithecopus azureus (Anura: Hylidae) in the Brazilian Pantanal. J Helminthol 2024; 98: e62, 1–5. https://doi.org/10.1017/S0022149X24000609.

Sokolov SG, Malysheva SV. Molecular characterization of *Ichtyobronema hamulatum* (Moulton, 1931) (Nematoda: Quimperiidae), a common parasite of burbot *Lota lota* (Linnaeus) (Actinopterygii: Lotidae). *Helminthologia* 2017; 54(3): 183–188.

Tran BT, Sato H, Luc PV. A new *Cosmocercoides* species (Nematoda: Cosmocercidae), *C. tonkinensis* n. sp., in the scale-bellied tree lizard (*Acanthosaura lepidogaster*) from Vietnam. *Acta Parasitol* 2015; 60(3): 407–416. https://doi.org/10.1515/ap-2015-0056.
